# The impact of physical therapy on dysphagia in neurological diseases: a review

**DOI:** 10.3389/fnhum.2024.1404398

**Published:** 2024-06-06

**Authors:** Kun Li, Cuiyuan Fu, Zhen Xie, Jiajia Zhang, Chenchen Zhang, Rui Li, Caifeng Gao, Jiahui Wang, Chuang Xue, Yuebing Zhang, Wei Deng

**Affiliations:** ^1^Shandong Daizhuang Hospital, Jining, China; ^2^Department of Psychology, Xinxiang Medical University, Xinxiang, China; ^3^Affiliated Mental Health Center and Hangzhou Seventh People’s Hospital, Zhejiang University School of Medicine, Hangzhou, China; ^4^Liangzhu Laboratory, MOE Frontier Science Center for Brain Science and Brain-machine Integration, State Key Laboratory of Brain-Machine Intelligence, Zhejiang University, Hangzhou, China

**Keywords:** Parkinson’s disease, stroke, schizophrenia, dysphagia, neuromuscular electrical stimulation, repetitive transcranial magnetic stimulation, sensory stimulation, transcranial direct current stimulation

## Abstract

A neurogenic dysphagia is dysphagia caused by problems with the central and peripheral nervous systems, is particularly prevalent in conditions such as Parkinson’s disease and stroke. It significantly impacts the quality of life for affected individuals and causes additional burdens, such as malnutrition, aspiration pneumonia, asphyxia, or even death from choking due to improper eating. Physical therapy offers a non-invasive treatment with high efficacy and low cost. Evidence supporting the use of physical therapy in dysphagia treatment is increasing, including techniques such as neuromuscular electrical stimulation, sensory stimulation, transcranial direct current stimulation, and repetitive transcranial magnetic stimulation. While initial studies have shown promising results, the effectiveness of specific treatment regimens still requires further validation. At present, there is a lack of scientific evidence to guide patient selection, develop appropriate treatment regimens, and accurately evaluate treatment outcomes. Therefore, the primary objectives of this review are to review the results of existing research, summarize the application of physical therapy in dysphagia management, we also discussed the mechanisms and treatments of physical therapy for neurogenic dysphagia.

## 1 Introduction

There are several complex physiological movements involved in swallowing, including movements of the mouth, pharynx, larynx, and esophagus (Shaw and Martino, 2013). Dysphagia refers to the disruption of the normal swallowing process (Rofes et al., 2011), which poses severe risks including malnutrition, aspiration pneumonia, asphyxia, etc (Hurtte et al., 2023). The causes of dysphagia can be divided into neurogenic, structural, and mental dysphagia (Medicine DRCO, 2023). A neurogenic dysphagia results from problems with the central and peripheral nervous systems (El Halabi et al., 2023). The number of people suffering from neurogenic dysphagia each year worldwide is estimated at 400000 to 800,000 (Panebianco et al., 2020). Among the diseases that predispose to neurogenic dysphagia are stroke, Parkinson’s disease, amyotrophic lateral sclerosis, multiple sclerosis, and other forms of neurodegeneration (Chandran and Doucet, 2024). It is a common complication of stroke to experience dysphagia afterward, it is estimated that 20–43% of patients have persistent dysphagia after 3 months, which can lead to aspiration pneumonia, malnutrition, water and electrolyte disorders, and other complications (Chen et al., 2024). There is a prevalence of 18–100% of Parkinson’s disease with dysphagia, it can lead to dehydration, malnutrition, aspiration pneumonia, depression, and social isolation, and it can also affect the quality of life and even cause death (Dashtelei et al., 2024). Dehydration, malnutrition, asphyxia, and death are all risks associated with neurogenic dysphagia, which severely reduces the quality of life for the patient (Xia et al., 2023; Kocica et al., 2024).

Physical therapy, as a new treatment method, directly targets the swallowing nerve circuit to enhance swallowing function (Li et al., 2023). Common clinical treatments for dysphagia include neuromuscular electrical stimulation (NMES), sensory stimulation, repetitive transcranial magnetic stimulation (rTMS), and transcranial direct current stimulation (tDCS) (Alvarez-Berdugo et al., 2016; Miller et al., 2022; Li et al., 2023). However, there remains paucity of discourse on the application of physical therapy in dysphagia management in different diseases. The purpose of this review article is to provide more theoretical support for the application of physical therapy in neurogenic dysphagia, and to describe treatment principles and treatment programs of several commonly used physical therapy programs.

## 2 Mechanisms associated with dysphagia

**Figure 1:**
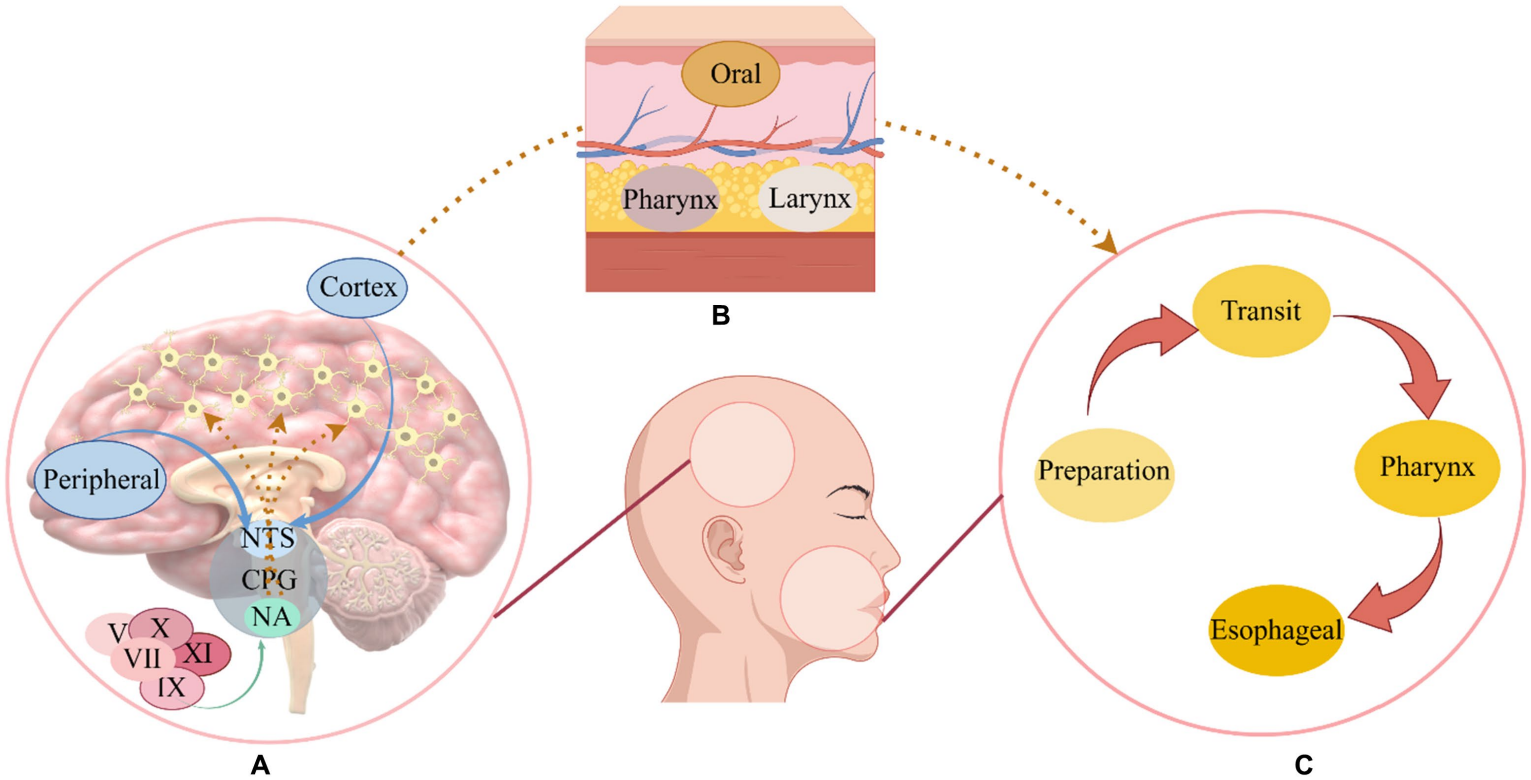
The mechanism of swallowing. As part of the swallowing central program generator, the nucleus ambiguous receives relevant cranial nerve stimulation and the nucleus tractus solitarius receives cortical and peripheral sensory input, after receiving the signal, the swallowing-related muscles are stimulated to promote the normal swallowing process (Yamamoto et al., 2022). **(A)** The swallowing center’s operation. **(B)** Swallowing muscles. **(C)** A normal swallowing process; NTS, Nucleus tractus solitarius; CPG, Central program generator; NA, Nucleus ambiguous; V, Trigeminal nerve; VII, Facial nerve; IX, Glossopharyngeal nerve; X, Vagus nerve; XI, Accessory nerve.

Swallowing is a complex process, it involves the coordination of more than 30 muscles in the mouth, pharynx, larynx, and esophagus, encompassing four distinct stages: oral preparation, oral transit, pharynx, and esophageal phase (Dodds et al., 1990). It involves various levels of the central nervous system, from the cortex to the medulla, as well as multiple cranial and peripheral nerves (El Halabi et al., 2023). It is well recognized that pharyngeal movements are strongly related to the innervation of sensory branches of the cranial nerves (Nascimento et al., 2021). Usually, swallowing is controlled by four types of components: (1) afferent motor fibers in cranial nerves and ansa cervicalis; (2) afferent sensory fibers in cranial nerves; (3) fibers lining the cerebral, cerebellar, and cerebellar hemispheres that synapse in the swallowing centers; (4) paired swallowing centers in the brainstem that synapse with each other (Dodds et al., 1990). In the swallowing process, fiber transmitters transmit signals from peripheral nerves and the cerebral cortex to the swallowing centers in the brain stem (Hashimoto et al., 2019). There is a complex unit called the swallowing central pattern generator that is composed of motor neurons and interneurons located in the brainstem’s medulla oblongata, a region that contains swallowing neurons (Sasegbon et al., 2024). Two parts make up the pattern generator of the swallowing center: (1) the dorsal region consisting of the nucleus tractus solitarius and peripheral neurons; (2) the nucleus and reticular formation surrounding the nucleus are located in the ventral region (Jang and Kim, 2021). Nucleus ambiguous innervates the muscles of the oral cavity, larynx, and pharynx through the trigeminal, facial, glossopharyngeal, vagus, and accessory nerves (Petko and Tadi, 2023; Chandran and Doucet, 2024), The nucleus tractus solitary can receive incoming information from the nucleus doubtful and then send efferent fibers to the corresponding muscles, but mainly integrates information from higher cortical centers and peripheral sensory afferents and regulates swallowing according to the nature of the food bolus (Chandran and Doucet, 2024; Ye et al., 2024). Overall (see Figure 1), the swallowing central pattern generator is responsible for the formation and regulation of swallowing motor sequences, processing incoming information, generating preprogrammed swallowing responses, and distributing appropriate signals to the motor nuclei of cranial nerves and their axons, which are ultimately transmitted to the many muscles involved in swallowing (Yamamoto et al., 2022).

A trigeminal and facial nerve innervate the muscles of the mouth, masticatory muscles are innervated by the trigeminal nerve, while the glossopharyngeal nerve and the vagus nerve innervate the muscles of the pharynx (Costa, 2018). In addition, the contraction of the esophageal sphincter affects the operation of swallowing, it contains the cricopharyngeal and hypopharyngeal constrictors and is innervated by the vagus nerve, while the muscularis that promote the contraction of the esophageal sphincter during swallowing, such as the suprahyoid muscles (stylohyoid, digastric, and mylohyoid muscles) and thyrohyoid muscles, are innervated by the trigeminal, facial, and hypoglossal nerves (McCarty and Chao, 2021). The recruitment of muscles necessary for the swallowing sequence is directed by the swallowing network, and integration between descending signals and afferent inputs may occur in the cortex and cerebellum, where there are multiple synaptic connections for different functions, the cerebral cortex may be responsible for the initiation of motor commands, and some cortical areas may be responsible for the integration of chewing and swallowing information, other regions, however, feed the descending signal back to the brain stem with the sensation of the bolus moving along the swallowing channel (Cheng et al., 2022). Although movement is directed by the cortex, the cerebellum is also associated with movement and plays a key role in the balanced coordination of muscle movements (Roostaei et al., 2014). Influences the cortical swallowing module consisting of primary motor, auxiliary motor, primary sensory cortical areas, and cingulate gyrus (Sasegbon and Hamdy, 2023).

The term “neurogenic dysphagia” refers to dysphagia, or dysfunction of swallowing mechanisms, in patients who have suffered a neurologic insult or disease (Teismann et al., 2007). Such diseases include stroke, Parkinson’s disease, and multiple sclerosis, among other neurodegenerative disease processes (Chandran and Doucet, 2024). Dysphagia following stroke primarily stems from cerebral cortex and subcortical structures damage, affecting areas like the motor cortex, cerebellum, thalamus, and other parts, as well as sensory defects of the pharyngeal mucosa (Teismann et al., 2007; Qin et al., 2023). It is characterized by a delayed or absent swallowing reflex and a premature overflow of bolus (Labeit et al., 2023), pharyngeal food residues and pharyngeal motility disorders (Warnecke et al., 2021). A dysphagia caused by Parkinson’s disease is different from dysphagia caused by stroke because it is primarily caused by problems with the brainstem, muscle atrophy, and dopaminergic and non-dopaminergic mechanisms (Patel et al., 2020). The patient presented with hypoesthesia of the pharynx, food residue, and bradykinesia of the oropharynx (Labeit et al., 2020a), the swallowing reflex was impaired, and the bolus overflowed prematurely (Labeit et al., 2020b). The pathological mechanisms of dysphagia in multiple sclerosis include damage of cortical bulbar fibers alone or in combination, damage of the brainstem swallowing center, abnormalities of the cerebellum affecting the accuracy of sequential planning and coordination of swallowing, failure of the afferent nerve central sensory pathway and abnormal impairment of the central motor pathway (Alfonsi et al., 2013). Its dysfunction may occur at any stage of swallowing and cause various complications such as aspiration pneumonia, malnutrition and airway obstruction (Ansari et al., 2020).

## 3 Physical therapy

### 3.1 Neuromuscular electrical stimulation

The purpose of NMES is to stimulate peripheral nerves associated with paralyzed pharyngeal muscles with low-frequency electrical stimulation, aiming to enhance their functionality (Doucet et al., 2012). In simpler terms, the effects of NMES on swallowing are improved through the contraction of pharyngeal muscles (Carnaby et al., 2020). Laryngeal elevation and reduction resulting from pharyngeal muscle defects are the primary causes of dysphagia in stroke patients, leading to potential issues such as aspiration and pharyngeal residue (Bath et al., 2018). Therefore, the NMES therapy is considered one of the most effective treatments for dysphagia caused by stroke (Beom et al., 2011). Moreover, patients with Parkinson’s disease often use NMES as a form of physical therapy to improve tongue muscle weakness (Park et al., 2018).

Preliminary studies indicated that increased tongue power results in greater activation of the suprahyoid muscle during swallowing (Oh, 2016). This goal can be achieved by NMES, by depolarizing motor axons and activation of type II fast-twitch muscle fibers in neuromuscular tissues, either through peripheral nerves or muscle belly (Carnaby et al., 2020; Carson and Buick, 2021). In patients with brain injury-related dysphagia, the NMES strengthens both suprahyoid and infrahyoid muscles, along with the muscles that assist in swallowing (Seo et al., 2021). Moreover, the long-term application of NMES benefits the recovery of swallowing-related cortical neuroplasticity in stroke patients (Zhang et al., 2022). Given the loss of swallowing motor control in stroke patients, functional muscle contraction patterns are primarily re-educated during NMES (Miller et al., 2022). This entails triggering the peripheral neuromuscular system via external electrical stimulus, depolarizing cervical muscle nerve fibers, and initiating oropharyngeal muscle contraction to improve swallowing function (Wang et al., 2023). In addition, it shows promise in improving dysphagia associated with other diseases such as Parkinson’s disease and head and neck cancer, in similar ways to how NMES improves dysphagia associated with strokes, it stimulates the nerve and motor endplates of the nerve (Tan et al., 2013).

In the application of NMES, electrodes are typically positioned on the hyoid muscles or adjacent areas, utilizing a frequency range of 25 to 120 Hz (Miller et al., 2022). Recent research findings, as summarized in Table S1, suggest the optimal treatment duration for NMES is generally between 20 to 30 min, with an application frequency of 80 Hz, and electrodes placed on the hyoid muscles (Miller et al., 2022). At present, studies consistently demonstrate that NMES improves swallowing function in patients suffering from neurogenic dysphagia, attributed to the following factors: (1) stimulation of the tongue, orbicular oris muscles, and other related muscles to promote the development of normal movement patterns and enhance organ and muscle functions; (2) alteration in the excitability of the pharyngeal cortex to promote normal swallowing mode operation; (3) activation of the swallowing center, facilitating the functional reconstruction of the nervous system (Konecny and Elfmark, 2018; Meng et al., 2018; Zeng et al., 2018; Oh et al., 2020).

**Figure 2:**
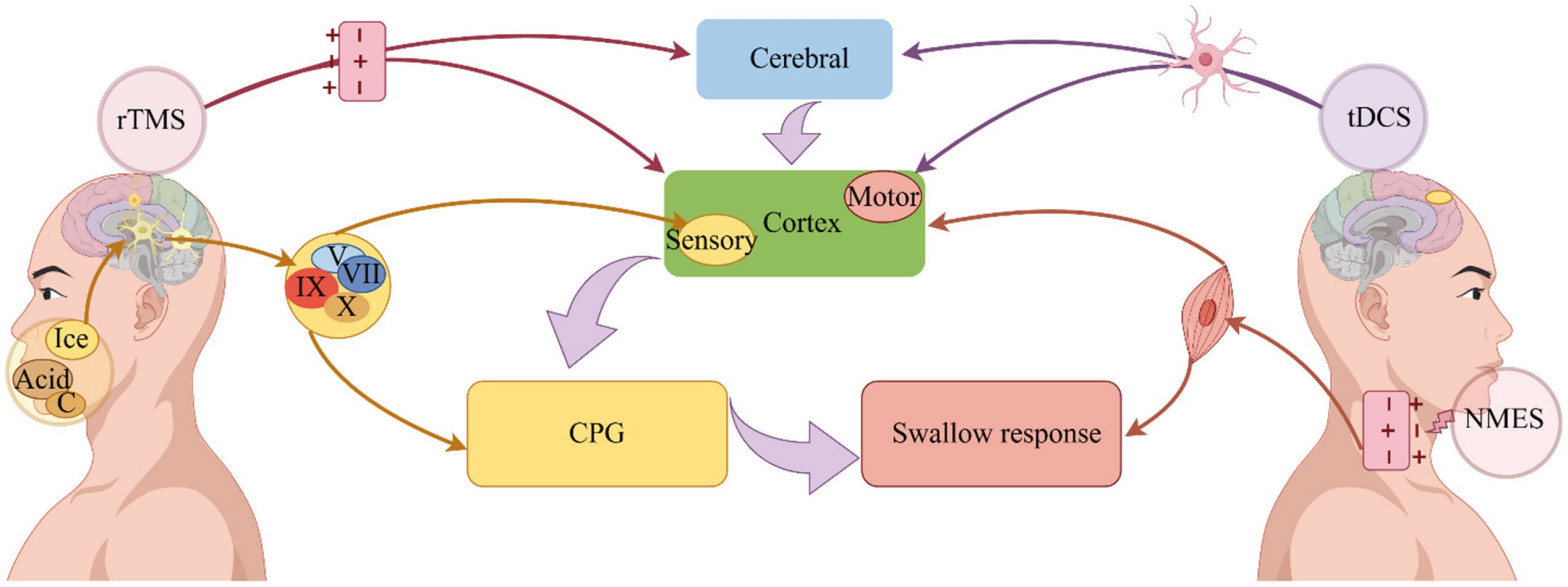
Improvement of dysphagia by physical therapy. Depolarization of axons is induced by NMES, which induces swallowing muscle contraction and improves swallowing (Bergquist et al., 2011). On the other hand, it can stimulate the excitability of the pharyngeal cortex, induce the operation of the central pattern generator, and improve swallowing (Zeng et al., 2018). Sensory stimulation transmits signals through the cranial nerves, on the one hand, directly to the central pattern generator, and on the other hand, it transmits signals to the cerebral cortex, where it is integrated and organized, and finally promotes the swallowing response (Alvarez-Berdugo et al., 2016). A change in neural plasticity is used to accelerate the operation of swallowing circuits through rTMS, which uses electromagnetic induction to depolarize synapses, ultimately improving swallowing (Labeit et al., 2024). Dysphagia can be improved by tDCS because it alters nerve cell polarity and triggers neuroplasticity (Speyer et al., 2022). NMES: Neuromuscular electrical stimulation; tDCS, Transcranial direct current stimulation; rTMS, Repetitive transcranial magnetic stimulation, CPG, Central pattern generator; C, Carbonation.

In summary (see Figure 2), the NMES stimulates the depolarization of the axons below the electrode, and the depolarization signal of motor axons propagates from the stimulation site to the muscle (peripheral pathway) to produce contraction, which can induce the neural plasticity of the central nervous system and enhance the neuromuscular function after nervous system injury (Bergquist et al., 2011). A NMES consists of muscle reeducation primarily focused on improving swallowing function by facilitating normal swallowing mode operation (Jeon et al., 2020).

### 3.2 Sensory stimulation

Sensory stimulation plays a crucial role in promoting the rehabilitation of swallowing function (Cola et al., 2012). Normal swallowing relies on somatosensory inputs associated with trigeminal, glossopharyngeal, and vagus nerves (Jean, 2001). When the sensory information pathway is impaired, reduced sensory input can slow down the swallowing-cortical pathway, resulting in dysphagia (Teismann et al., 2007). Sensory stimulation of the cranial nerves enhances the transmission of information to the solitary tract nucleus in the brainstem (Alvarez-Berdugo et al., 2016). This, in turn, increases the sensory input to the nucleus tract solitary in the brainstem through cranial nerves, promoting the operation of normal swallowing pattern, and ultimately improving swallowing function (Jean, 2001). Common sensory stimulation methods include ice, acid, and carbonation stimulation (Regan, 2020).

Ice stimulation therapy uses repetitive mechanical, pressure, and temperature stimulation to enhance the sensitivity of the soft palate and pharynx. By increasing the sensory sensitivity of local nerves, ice stimulation prompts local muscles contraction and triggers the swallowing reflex (Li et al., 2017). Consequently, ice stimulation mobilizes resting neuron excitability, reconstructs the neural network to achieve functional reorganization, promotes the normal swallowing reflex, and restores the function of swallowing organs (Ilott et al., 2016). In the application of ice stimulation therapy Nakamura and Fujishima (2013) dipped a cotton stick about 10 cm long and 1.27 cm in diameter into the water until it froze into a frozen sucker, then lightly rubbed and pressed it against the posterior tongue, bottom of the tongue, and posterior pharyngeal wall of stroke patients with dysphagia for 10 s, results revealed that ice stick massage could shorten the threshold of the swallowing response phase. Kawakami et al. (2019) demonstrated that placing an ice stick in the mouth is superior to placing it on the neck. Intraoral ice stimulation significantly can increase the excitability of the swallowing pathway in the cortex, trigger swallowing initiation, and shorten the duration of the pharyngeal phase. Early rehabilitation of stroke patients with dysphagia is closely related to the central nervous system’s ability to compensate and reconstruct injured areas, thereby increasing the excitability of the nervous system and facilitating swallowing by activating the central nervous system to form new sensory and motor projections (Qin et al., 2019). At this point, when applied to stroke-related dysphagia, ice stimulation’s effects primarily manifest in two ways. Firstly, it activates sensory nerve fibers, boosts sensory recovery, and restores the neural network (Ferrara et al., 2018). Secondly, it enhances sensory sensitivity. By increasing the sensitivity of the swallowing reflex area, it amplifies sensory inputs before swallowing, induces the generation of swallowing reflex, and finally improves swallowing function (Cui et al., 2020).

The improvement of dysphagia through acid stimulation may be attributed to sensory feedback information (Logemann et al., 1995). Previous studies investigating neurogenic swallowing dysfunction found that subjects consuming water with a citric acid concentration of 2.7%, compared to plain water, exhibited increased spontaneous swallowing and reduced aspirations, leading to an improvement in swallowing function (Pelletier and Lawless, 2003). Wang et al. (2022) reported an effective acid stimulation medium for treating dysphagia in stroke patients. This method entailed applying vitamin C tablet powder (0.2 g/day) to the patient’s bilateral tongue using a cotton swab, followed by swallowing practice instructions. Additionally, tongue massage with the cotton swab and guidance for tongue and masticatory muscle exercises were included (5–6 times per day, 15 min each time for 2 weeks). Acid stimulation promotes saliva secretion by stimulating the tongue, thereby accelerating the swallowing process and relieving swallowing disorders (Wang et al., 2022). Acid stimulation effectively improves stroke dysphagia based on two fundamental principles. Furthermore, acid stimulation increases the activity intensity of swallowing-related muscles such as the mylohyoid and front belly of the digastric muscles, triggering stronger contractions during swallowing and consequently improving dysphagia (Palmer et al., 2005).

Carbonation stimulation is also considered to be a beneficial sensory stimulation technique for improving dysphagia, primarily by enhancing the contractility of the velum and oropharynx (Omari et al., 2020) as well as prolonging the opening duration of the upper esophageal sphincter (Miura et al., 2009). A recent study conducted by Morishita et al. (2023) has suggested that carbonation may induce changes in brain excitability, resulting in shorter swallowing times in healthy individuals consuming carbonated beverages. Additionally Bülow et al. (2003) demonstrated that carbonation stimulation effectively reduces airway aspiration and pharyngeal retention, and shortens the duration of the pharyngeal phase duration. Sdravou et al. (2012) conducted an experiment involving carbonation stimulation in 17 patients with neurogenic dysphagia, confirming that drinking carbonated water can reduce aspiration. The effectiveness of carbonation in improving swallowing function can be attributed to two main factors. On the one hand, it is related to the activation of swallowing pathways. Stimulation of peripheral sensory receptors and sensory fibers in the nucleus tractus solitarius in the brainstem activates the pattern generator of the swallowing center (Nagano et al., 2022). On the other hand, carbonation affects numerous receptors in the larynx, namely mechanoreceptors, chemoreceptors, pain receptors, and thermoreceptors, which respond to carbonation stimulation by triggering protective reflexes to prevent aspiration (Bradley, 2000).

In summary (see Figure 2), the intensity and duration of the swallowing response can be triggered or regulated by a complex biofeedback mechanism, sensory stimulation transmit signals mainly through the trigeminal, facial, glossopharyngeal and vagus nerves, which on the one hand directly reach the swallowing central pattern generator, and on the other hand, transmit signals to the cerebral cortex, which outputs information to the swallowing central pattern generator for integration and tissue, the swallowing response is facilitated (Alvarez-Berdugo et al., 2016).

### 3.3 Repetitive transcranial magnetic stimulation

The technique of rTMS involves placing a coil to the head to generate a magnetic field when an electric current passes through it, this magnetic field induces current flows within brain tissue perpendicular to its direction, which are of strong strength to induce modifications in both cortical and subcortical white matter axons (Ridding and Rothwell, 2007). The rTMS can either suppress or excite neuronal activity depending on the frequency used: frequencies at or below 1 Hz suppress neuronal activity, while those above 5 Hz elicit neuronal excitation (Honda et al., 2021). Studies on healthy participants have investigated the effects of rTMS on the pharyngeal motor cortex (Yamamura et al., 2018). Findings indicate that rTMS at 1 Hz inhibits the excitability of the pharyngeal motor cortex, whereas high-frequency stimulation, such as 10 Hz stimulation of the cerebellar hemisphere increases the amplitude of pharynx cortical motor evoked potentials (Vasant et al., 2015; Sasegbon et al., 2020b). Due to its potential neural repair mechanisms, dysphagia has been treated extensively with rTMS (Sasegbon et al., 2020a). Recent schemes for rTMS treatment of dysphagia are summarized in Table S2. According to Table S2, treatment with rTMS focuses primarily on the cerebellum and pharyngeal motor cortex. High frequencies are predominantly used for treatment frequency, and the treatment time is mostly selected daily, 5 days a week, for a total of 2 weeks, and the swallowing function test results are improved (Dong et al., 2022; Rao et al., 2022; Zhong et al., 2023).

There is evidence that rTMS alters cortical excitability, regulates neurotransmitter release, and promotes neuroplasticity in the brain (Kesikburun, 2022). The increase in cortical activity in the cerebral hemispheres is associated with functional recovery in stroke patients with dysphagia, and the reorganization of neural networks plays a significant role in the recovery of swallowing function (Hoogendam et al., 2010). The changes in neuroplasticity are closely related to rTMS induced synaptic connections in the process of regulating the functional state of the cerebral cortex (Li et al., 2022). There is no single target for rTMS treatment of stroke dysphagia; rather, it involves the regeneration of swallowing function in stroke patients through the cooperative action of multiple brain areas (Dong et al., 2022). After virtual lesion simulation in stroke patients with dysphagia, cerebellar high frequency rTMS not only improves the excitability of the pharyngeal motor cortex in healthy volunteers (Sasegbon et al., 2019, 2020b) but also improves swallowing function among stroke patients with dysphagia (Zhong et al., 2023). This may be explained by the fact that the cerebellum is connected to the brainstem by three cerebellar peduncles, which directly communicate with the various motor nuclei of the brainstem (Roostaei et al., 2014). Based on the evidence that rTMS could improve not only swallowing disorder but also motor function, Khedr et al. applied rTMS to Parkinson’s disease patients with dysphagia and achieved the envisaged results: Parkinson’s disease patients could benefit from rTMS for dysphagia (Khedr et al., 2019).

In summary (see Figure 2), the cerebral cortex and cerebellum are the primary stimulation targets for rTMS in dysphagia, it uses electromagnetic induction to depolarization synapses and accelerate the operation of swallowing circuit through changes in neuroplasticity, so as to improve swallowing function (Speyer et al., 2022; Labeit et al., 2024).

### 3.4 Transcranial direct current stimulation

The tDCS technique is a groundbreaking method of non-invasive brain stimulation (Pisegna et al., 2016) that involves applying small electrical currents (1–2 mA) through two surface electrodes, the anode electrode and cathode electrode, to targeted brain regions, thereby triggering and modulating brain activity (He et al., 2022). In recent years, there has been strong interest in tDCS as an effective, noninvasive method to treat dysphagia (Cheng et al., 2021). Table S3 shows the application of tDCS in dysphagia treatment in recent years. It can be seen from the table that tDCS is mostly used for stroke-related dysphagia. The location of the anode is related to the area involved in the pharyngeal motor cortex. The cathodes are mostly placed in the contralateral supraorbital region and the opposite shoulder, most single intervention sessions last 20 min, the treatment effect can generally achieve the improvement of swallowing function (Farpour et al., 2023; El Nahas et al., 2024).

Neuroplasticity is the concept behind tDCS, which is a form of noninvasive brain stimulation (Kesikburun, 2022). The swallowing motor task-related activities of the brain are enhanced through glutamatergic and calcium-dependent processes, including synaptogenesis, reorganization, strengthening, and inhibition of brain networks (Pisegna et al., 2016; Tedesco Triccas et al., 2016). Anodal tDCS can improve swallowing function by stimulating the pharyngeal motor cortex in patients with Parkinson’s disease, which is associated with an increase in the strength of synaptic connections related to deglutition in the cerebral cortex (Tedesco Triccas et al., 2016). Dysphagia can also be ameliorated by tDCS in older adults with and without neurological conditions, which is associated with the induction of a polar-dependent shift in underlying cortical excitability, as well as a broad activation of pharyngeal motor cortex in both brain hemispheres (Cosentino et al., 2020). Furthermore, stroke patients often choose tDCS as a treatment for dysphagia, with numerous therapeutic targets (Gómez-García et al., 2023), related to the regions involved in the swallowing network and the fact that tDCS promotes neuroplasticity (Ahn et al., 2017). For example, enhancing the excitability of the uninjured side of the swallowing cortex, the injured side of the swallowing cortex, the bilateral swallowing cortex, and the superior limbic gyrus can be beneficial for enhancing swallowing function (Wang et al., 2020; Mao et al., 2022; Farpour et al., 2023). Dysphagia in stroke patients can be effectively improved by tDCS, and anodal stimulation of the right swallowing cortex in patients with multiple sclerosis dysphagia can also improve swallowing function, as anodal stimulation of the swallowing motor cortex of the right also activates an extensive network involving the contralateral hemisphere to compensate for the damage caused by focal brain injury (Cosentino et al., 2018).

In general (see Figure 2), the treatment principle of tDCS can improve dysphagia in a variety of diseases (Lefaucheur, 2016). Its current directly stimulates the brain or cerebellum and changes the polarity of nerve cells, aiming to trigger and promote neuroplasticity and improve dysphagia (Speyer et al., 2022; Labeit et al., 2024).

## 4 Summary and prospect

Neurogenic dysphagia is currently managed with motor training, oral medications, and surgery (El Halabi et al., 2023). Clinical evidence supports the use of movement training for mild-to-moderate dysphagia (Saconato et al., 2016), with strong recommendations from intermediate and high-level evidence sources (Medicine DRCO, 2023; Yang et al., 2023). Patients with severe dysphagia typically undergo medical or surgical interventions, especially at intermediate and advanced disease stages (Cotaoco et al., 2024). While conventional treatments may take longer to reach full efficacy, physical therapy has emerged as a non-invasive and practical approach to shorten treatment duration for dysphagia patients (Frost et al., 2018; Bengisu et al., 2024). This review elucidates the physiological function of the swallowing system, the pathological mechanisms of neurogenic dysphagia, and the principles and application of physical therapy. Overall, physical therapy offers benefits to individuals with neurogenic dysphagia by enhancing swallowing recovery, improving treatment outcomes, and enhancing quality of life (Banda et al., 2023; Bengisu et al., 2024).

Considering the increasing public interest in neurogenic dysphagia, it is crucial to also address its effects on individuals with schizophrenia. Future research should prioritize investigating the pathogenesis of dysphagia in schizophrenia, exploring the effectiveness of physical therapy interventions, and identifying the most suitable therapy targets for this specific population.

## Author contributions

KL: Conceptualization, Writing – review & editing, Funding acquisition, Supervision. CF: Writing – review & editing, Conceptualization, Writing – original draft. ZX: Writing – review & editing. JZ: Writing – original draft. CZ: Investigation, Writing – original draft. RL: Investigation, Writing – original draft. CG: Supervision, Writing – review & editing. JW: Writing – original draft. CX: Writing – review & editing, Investigation. YZ: Writing – review & editing. WD: Writing – review & editing.
