# Supplementary material for: The impact of physical therapy on dysphagia in neurological diseases: a review
Source: Front Hum Neurosci. 2024 Jun 6;18:1404398. doi: 10.3389/fnhum.2024.1404398 (PMC11187312; doi:10.3389/fnhum.2024.1404398)
Supplement: Supplementary file 1 [file Data_Sheet_1.docx]

Supplementary Material

**Supplementary Table 1. NMES treatment protocol for dysphagia**

| Study | Subjects | N | Frequency | Time | Stimulation  target | Outcome  measures | Major outcomes |
| --- | --- | --- | --- | --- | --- | --- | --- |
| Oh et al (Oh et al., 2020) | Stroke | 38 | 80 Hz | 30 min each time, 5 d/W, 4 W | Left: suprahyoid; Right: infrahyoid | VFSS, VDS, PAS, FOIS | SMG did not show significant changes in any of the measures compared to IMG |
| Sproson et al (Sproson et al., 2018) | Stroke | 30 | 30 Hz | 30 min each time, 5 d/W, 4 W | Suprahyoid muscles | FOIS, PAS, SWAL-QoL | Active >Control: ↑FOIS, ↓PAS, ↑SWAL-QoL |
| Konecny et al (Konecny and Elfmark, 2018) | Stroke | 108 | 60 Hz | 20 min each time, 5 d/W, 4 W | Suprahyoid  muscles | VFSS | Study >Control：  NMES significantly reduced swallowing and improved swallowing |
| Meng et al (Meng et al., 2018) | Stroke | 30 | 80 Hz | 30 min each time, 5 d/W, 2 W | TGA: suprahyoid, thyroid cartilage. TGB: geniohyoid, mylohyoid | VFSS, WST, RSST, DOSS | TGA, TGB >Control：  ↑VFSS, ↓WST, ↓RSST, ↑DOSS |
| Simonelli et al (Simonelli et al., 2019) | Stroke | 33 | 80 Hz | 30 min each time, two times per day, 5 d /W, 8 W | Suprahyoid  muscles | FOIS, PAS, FEES | NMES+TDT >TDT: ↑FOIS, ↓PAS, ↑FEES |

**Supplementary Table 2. Summary of rTMS treatment regimens**

| Study | Subjects | | N | rTMS Methods | Time | Stimulation  target | Outcome  measures |
| --- | --- | --- | --- | --- | --- | --- | --- |
| Rao et al (Rao et al., 2022) | | Stroke | 70 | iTBS, 100% RMT, 600 pulses each | Five days a week, two weeks | The pharyngeal motor cortexof the cerebellum | FEDSS, WST, PAS, SSA, FOIS |
| Dong et al (Dong et al., 2022) | | Stroke | 36 | 10 Hz, 80% RMT, 250 pulses | Five days a week, two weeks | The pharyngeal motor cortex of the cerebellum | FDS, PAS |
| Du et al (Du et al., 2022) | | Cerebral infarction | 180 | 80% MT, 600 pulses | 30 days | The Cz point was 3 cm forward and 8 cm toward the infarct side | Kubota’s water swallow test, VFSS |
| Jiao et al (Jiao et al., 2022) | | Stroke | 61 | 3 Hz , 80% MT, 1600 pulses | Five days a week, two weeks | The pharyngeal motor cortexof the lesioned hemisphere | WST |
| Park et al (Park et al., 2017) | | Stroke | 35 | 10 Hz, 90% MT, 500 pulses each | 14 days | Pharyngeal motor cortex | CDS, DOSS, PAS, VDS |
| Zou et al (Zou et al., 2023) | | Stroke | 45 | High:  5 Hz, 90% RMT, 600 pulses each  Low:  1 Hz, 120% RMT, 600 pulses each | Five days a week, four weeks | The pharyngeal motor cortex of the hemisphere | DHI, FOIS, PAS |
| Zhong et al (Zhong et al., 2023) | | Stroke | 85 | 10 Hz, 80% RMT, 250 pulses each | Five days a week, two weeks | The pharyngeal motor cortex of the cerebellum | FEDSS, PAS |
| Khedr et al(Khedr et al., 2019) | | Parkinson | 33 | 20 Hz, 90% RMT, 2000 pulses each | Five days a week, two weeks | Hemisphere (right then left hemisphere) | A-DHI, PTT |

**Supplementary Table 3. Summary of tDCS treatment regimens**

| Study | Subjects | N | Electrode position | | Parameters | Time | Major outcomes |
| --- | --- | --- | --- | --- | --- | --- | --- |
|  |  |  | Anode (position in the International System of Electrodes) | Cathode (reference electrode) |  |  |  |
| El Nahas et al. (El Nahas et al., 2024) | Stroke | 48 | Left pharyngeal motor cortex | Right shoulder | 1.5 mA, 20 min | Daily for six sessions | tDCS / NMES >Sham, NMES: GUSS, FEES |
| Farpour et al. (Farpour et al., 2023) | Stroke | 48 | Intact supra marginal gyrus | Contralateral supraorbital region | 2 mA, 20 min | 5 days | tDCS >Sham: FOIS, MASA |
| Wang et al. (Wang et al., 2023) | Stroke | 40 | The pharyngeal motor cortex of the unaffected side | Contralateral shoulder | 1 m A, 20 min | 5 times per week for 2 weeks | There was no significant difference between the two groups ：SSA, PAS |
| Mao et al. (Mao et al., 2022) | Stroke | 40 | The pharyngeal motor cortex on the nonlesional hemisphere | The opposite shoulder | 1.6 mA, 20 min | once a day, 6 days a week, 8 weeks. | Treatment >Conventional:  DOSS, FDS, VFSS |
| Sawan et al. (Sawan et al., 2020) | Stroke | 40 | Pharyngeal motor cortex | Contralateral supraorbital region | 3 A, 20 min | 5 times per week for 2 weeks | Study group >Control group: DOSS, VFS |
| Wang et al. (Wang et al., 2020) | Stroke | 28 | Hemispheres | Contralateral supraorbital region | 1 mA, 20 min | 20 days | tDCS >Sham: FDS, FOIS, PESO |
| Suntrup‑Krueger et al. (Suntrup-Krueger et al., 2018) | Stroke | 60 | Pharyngeal motor cortex | Contralateral supraorbital region | 1 mA, 20 min | 4 days | tDCS >Sham:  FEDSS, DSRS, FOIS |
| Ahn et al. (Ahn et al., 2017) | Stroke | 26 | Bilateral pharyngeal motor cortex | Contralateral supraorbital region | 2 mA, 20 min | 5 times per week for 2 weeks | tDCS >Sham: DOSS |

**References**

Ahn, Y.H., Sohn, H.-J., Park, J.-S., Ahn, T.G., Shin, Y.B., Park, M., et al. (2017). Effect of bihemispheric anodal transcranial direct current stimulation for dysphagia in chronic stroke patients: A randomized clinical trial. *Journal of Rehabilitation Medicine* 49(1)**,** 30-35. doi: 10.2340/16501977-2170.

Carnaby, G., LaGorio, L., Silliman, S., and Crary, M. (2020). Exercise-based swallowing intervention (McNeill Dysphagia Therapy) with adjunctive NMES to treat dysphagia post-stroke: A double-blind placebo-controlled trial. *Journal of oral rehabilitation* 47(4)**,** 501-510. doi: 10.1111/joor.12928.

Cheng, I.K.Y., Chan, K.M.K., Wong, C.-S., Li, L.S.W., Chiu, K.M.Y., Cheung, R.T.F., et al. (2017). Neuronavigated high-frequency repetitive transcranial magnetic stimulation for chronic post-stroke dysphagia: A randomized controlled study. *Journal of Rehabilitation Medicine* 49(6)**,** 475-481. doi: 10.2340/16501977-2235.

Cosentino, G., Gargano, R., Bonura, G., Realmuto, S., Tocco, E., Ragonese, P., et al. (2018). Anodal tDCS of the swallowing motor cortex for treatment of dysphagia in multiple sclerosis: a pilot open-label study. *Neurological sciences : official journal of the Italian Neurological Societyof the Italian Society of Clinical Neurophysiology* 39(8)**,** 1471-1473. doi: 10.1007/s10072-018-3443-x.

Cosentino, G., Tassorelli, C., Prunetti, P., Bertino, G., De Icco, R., Todisco, M., et al. (2020). Anodal transcranial direct current stimulation and intermittent theta-burst stimulation improve deglutition and swallowing reproducibility in elderly patients with dysphagia. *Neurogastroenterology and Motility* 32(5)**,** e13791. doi: 10.1111/nmo.13791.

Dashtelei, A.A., Nitsche, M.A., Bakhtiari, J., Habibi, S.A., Sepandi, M., and Khatoonabadi, A.R. (2020). The effects of spaced transcranial Direct Current Stimulation combined with conventional dysphagia therapy in Parkinson's disease: A case report. *EXCLI Journal* 19**,** 745-749. doi: 10.17179/excli2020-1453.

Dong, L., Pan, X., Wang, Y., Bai, G., Han, C., Wang, Q., et al. (2022). High-Frequency Cerebellar rTMS Improves the Swallowing Function of Patients with Dysphagia after Brainstem Stroke. *Neural plasticity* 2022**,** 6259693. doi: 10.1155/2022/6259693.

Du, J., Yang, F., Liu, L., Hu, J., Cai, B., Liu, W., et al. (2016). Repetitive transcranial magnetic stimulation for rehabilitation of poststroke dysphagia: A randomized, double-blind clinical trial. *Clinical Neurophysiology : Official Journal of the International Federation of Clinical Neurophysiology* 127(3)**,** 1907-1913. doi: 10.1016/j.clinph.2015.11.045.

Du, Y., Wei, L., Lu, Y., and Gao, H. (2022). The effects of different frequencies of repetitive transcranial magnetic stimulation (rTMS) on patients with swallowing disorders after cerebral infarction. *NeuroRehabilitation* 50(1)**,** 115-122. doi: 10.3233/nre-210176.

El Nahas, N., Shokri, H., Refaat, A., Mousa, H., Hamid, A., Abdel Monem, A., et al. (2024). The effect of transcranial direct current stimulation paired with neuromuscular electrical stimulation on swallowing function in post stroke dysphagia. *The Egyptian Journal of Neurology, Psychiatry and Neurosurgery* 60(1)**,** 21. doi: 10.1186/s41983-023-00767-8.

Farpour, S., Asadi-Shekaari, M., Borhani Haghighi, A., and Farpour, H. (2023). Improving Swallowing Function and Ability in Post Stroke Dysphagia: A Randomized Clinical Trial. *Dysphagia* 38(1)**,** 330-339. doi: 10.1007/s00455-022-10470-0.

Jiao, Y., Li, G., and Dai, Y. (2022). Clinical effect of repetitive transcranial magnetic stimulation on dysphagia due to stroke. *Neurological Sciences : Official Journal of the Italian Neurological Society and of the Italian Society of Clinical Neurophysiology* 43(5)**,** 3139-3144. doi: 10.1007/s10072-021-05799-7.

Khedr, E.M., Mohamed, K.O., Soliman, R.K., Hassan, A.M.M., and Rothwell, J.C. (2019). The Effect of High-Frequency Repetitive Transcranial Magnetic Stimulation on Advancing Parkinson's Disease With Dysphagia: Double Blind Randomized Clinical Trial. *Neurorehabilitation and Neural Repair* 33(6)**,** 442-452. doi: 10.1177/1545968319847968.

Konecny, P., and Elfmark, M. (2018). Electrical stimulation of hyoid muscles in post-stroke dysphagia. *Biomedical papers of the Medical Faculty of the University Palacky, Olomouc, Czechoslovakia* 162(1)**,** 40-42. doi: 10.5507/bp.2017.043.

Lee, S., Park, D., Jang, J., Jang, E., Lee, J., Park, Y., et al. (2021). Compensatory Effects of Sequential 4-Channel Neuromuscular Electrical Stimulation for the Treatment of Acute, Subacute, and Chronic Dysphagia in a Prospective, Double-Blinded Randomized Clinical Trial. *Neurorehabilitation neural repair* 35(9)**,** 801-811. doi: 10.1177/15459683211029891.

Li, X., and Li, L. (2019). Efficacy of neuromuscular electrical stimulation on Wilson's disease patients with dysphagia. *Journal of physical therapy science* 31(12)**,** 971-974. doi: 10.1589/jpts.31.971.

Mao, H., Lyu, Y., Li, Y., Gan, L., Ni, J., Liu, L., et al. (2022). Clinical study on swallowing function of brainstem stroke by tDCS. *Neurological sciences : official journal of the Italian Neurological Society of the Italian Society of Clinical Neurophysiology* 43(1)**,** 477-484. doi: 10.1007/s10072-021-05247-6.

Meng, P., Zhang, S., Wang, Q., Wang, P., Han, C., Gao, J., et al. (2018). The effect of surface neuromuscular electrical stimulation on patients with post-stroke dysphagia. *Journal of back musculoskeletal rehabilitation* 31(2)**,** 363-370. doi: 10.3233/bmr-170788.

Miller, S., Diers, D., Jungheim, M., Schnittger, C., Stürenburg, H., and Ptok, M. (2021). Studying effects of neuromuscular electrostimulation therapy in patients with dysphagia: which pitfalls may occur? A translational phase I study. *German medical science : GMS e-journal* 19**,** Doc07. doi: 10.3205/000294.

Oh, D., Park, J., Kim, H., Chang, M., and Hwang, N. (2020). The effect of neuromuscular electrical stimulation with different electrode positions on swallowing in stroke patients with oropharyngeal dysphagia: A randomized trial. *Journal of back musculoskeletal rehabilitation* 33(4)**,** 637-644. doi: 10.3233/bmr-181133.

Park, E., Kim, M., Chang, W., Oh, S., Kim, Y., Lee, A., et al. (2017). Effects of Bilateral Repetitive Transcranial Magnetic Stimulation on Post-Stroke Dysphagia. *Brain stimulation* 10(1)**,** 75-82. doi: 10.1016/j.brs.2016.08.005.

Park, J.-W., Kim, H., Park, T., Yeo, J.-S., Hong, H.-J., and Oh, J.-Y. (2019). A pilot study of the effects of high-frequency repetitive transcranial magnetic stimulation on dysphagia in the elderly. *Neurogastroenterology and Motility* 31(5)**,** e13561. doi: 10.1111/nmo.13561.

Park, J., Oh, D., Hwang, N., and Lee, J. (2018). Effects of neuromuscular electrical stimulation in patients with Parkinson's disease and dysphagia: A randomized, single-blind, placebo-controlled trial. *NeuroRehabilitation* 42(4)**,** 457-463. doi: 10.3233/nre-172306.

Rao, J., Li, F., Zhong, L., Wang, J., Peng, Y., Liu, H., et al. (2022). Bilateral Cerebellar Intermittent Theta Burst Stimulation Combined With Swallowing Speech Therapy for Dysphagia After Stroke: A Randomized, Double-Blind, Sham-Controlled, Clinical Trial. *Neurorehabilitation neural repair* 36(7)**,** 437-448. doi: 10.1177/15459683221092995.

Sawan, S.A.E., Reda, A.M., Kamel, A.H., and Ali, M.A.M. (2020). Transcranial direct current stimulation (tDCS): its effect on improving dysphagia in stroke patients. *The Egyptian Journal of Neurology, Psychiatry and Neurosurgery* 56(1)**,** 111. doi: 10.1186/s41983-020-00246-4.

Simonelli, M., Ruoppolo, G., Iosa, M., Morone, G., Fusco, A., Grasso, M., et al. (2019). A stimulus for eating. The use of neuromuscular transcutaneous electrical stimulation in patients affected by severe dysphagia after subacute stroke: A pilot randomized controlled trial. *NeuroRehabilitation* 44(1)**,** 103-110. doi: 10.3233/nre-182526.

Sproson, L., Pownall, S., Enderby, P., and Freeman, J. (2018). Combined electrical stimulation and exercise for swallow rehabilitation post-stroke: a pilot randomized control trial. *International journal of language communication disorders* 53(2)**,** 405-417. doi: 10.1111/1460-6984.12359.

Suntrup-Krueger, S., Ringmaier, C., Muhle, P., Wollbrink, A., Kemmling, A., Hanning, U., et al. (2018). Randomized trial of transcranial direct current stimulation for poststroke dysphagia. *Annals of neurology* 83(2)**,** 328-340. doi: 10.1002/ana.25151.

Tarameshlu, M., Ansari, N.N., Ghelichi, L., and Jalaei, S. (2019). The effect of repetitive transcranial magnetic stimulation combined with traditional dysphagia therapy on poststroke dysphagia: a pilot double-blinded randomized-controlled trial. *International Journal of Rehabilitation Research. Internationale Zeitschrift Fur Rehabilitationsforschung. Revue Internationale de Recherches de Readaptation* 42(2)**,** 133-138. doi: 10.1097/MRR.0000000000000336.

Ünlüer, N.Ö., Temuçin, Ç.M., Demir, N., Serel Arslan, S., and Karaduman, A.A. (2019). Effects of Low-Frequency Repetitive Transcranial Magnetic Stimulation on Swallowing Function and Quality of Life of Post-stroke Patients. *Dysphagia* 34(3)**,** 360-371. doi: 10.1007/s00455-018-09965-6.

Vasant, D.H., Sasegbon, A., Michou, E., Smith, C., and Hamdy, S. (2019). Rapid improvement in brain and swallowing behavior induced by cerebellar repetitive transcranial magnetic stimulation in poststroke dysphagia: A single patient case-controlled study. *Neurogastroenterology and Motility* 31(7)**,** e13609. doi: 10.1111/nmo.13609.

Wang, L., Shi, A., Xue, H., Li, Q., Wang, J., Yang, H., et al. (2023). Efficacy of Transcranial Direct Current Stimulation Combined with Conventional Swallowing Rehabilitation Training on Post-stroke Dysphagia. *Dysphagia* 38(6)**,** 1537-1545. doi: 10.1007/s00455-023-10581-2.

Wang, Z., Chen, J., Lin, Z., and Ni, G. (2020). Transcranial direct current stimulation improves the swallowing function in patients with cricopharyngeal muscle dysfunction following a brainstem stroke. *Neurological sciences : official journal of the Italian Neurological Society of the Italian Society of Clinical Neurophysiology* 41(3)**,** 569-574. doi: 10.1007/s10072-019-04120-x.

Zeng, Y., Yip, J., Cui, H., Guan, L., Zhu, H., Zhang, W., et al. (2018). Efficacy of neuromuscular electrical stimulation in improving the negative psychological state in patients with cerebral infarction and dysphagia. *Neurological research* 40(6)**,** 473-479. doi: 10.1080/01616412.2018.1451015.

Zhang, C., Zheng, X., Lu, R., Yun, W., Yun, H., and Zhou, X. (2019). Repetitive transcranial magnetic stimulation in combination with neuromuscular electrical stimulation for treatment of post-stroke dysphagia. *The Journal of International Medical Research* 47(2)**,** 662-672. doi: 10.1177/0300060518807340.

Zhang, Y., Dou, Z., Zhao, F., Xie, C., Shi, J., Yang, C., et al. (2022). Neuromuscular electrical stimulation improves swallowing initiation in patients with post-stroke dysphagia. *Frontiers in neuroscience* 16**,** 1011824. doi: 10.3389/fnins.2022.1011824.

Zhong, L., Wen, X., Liu, Z., Li, F., Ma, X., Liu, H., et al. (2023). Effects of bilateral cerebellar repetitive transcranial magnetic stimulation in poststroke dysphagia: A randomized sham-controlled trial. *NeuroRehabilitation* 52(2)**,** 227-234. doi: 10.3233/NRE-220268.

Zou, F., Chen, X., Niu, L., Wang, Y., Chen, J., Li, C., et al. (2023). Effect of Repetitive Transcranial Magnetic Stimulation on Post-stroke Dysphagia in Acute Stage. *Dysphagia* 38(4)**,** 1117-1127. doi: 10.1007/s00455-022-10533-2.
